# Heat and Drought Stress Impact on Phenology, Grain Yield, and Nutritional Quality of Lentil (*Lens culinaris* Medikus)

**DOI:** 10.3389/fnut.2020.596307

**Published:** 2020-11-23

**Authors:** Hasnae Choukri, Kamal Hejjaoui, Adil El-Baouchi, Noureddine El haddad, Abdelaziz Smouni, Fouad Maalouf, Dil Thavarajah, Shiv Kumar

**Affiliations:** ^1^Laboratoire de Biotechnologie et de Physiologie Végétales, Faculté des Sciences, Centre de Recherche BioBio, University Mohammed V in Rabat, Rabat, Morocco; ^2^International Center for Agricultural Research in the Dry Areas (ICARDA), Rabat, Morocco; ^3^International Center for Agricultural Research in the Dry Areas (ICARDA), Terbol, Lebanon; ^4^Plant and Environmental Sciences, Pulse Quality and Organic Breeding, Clemson University, Clemson, SC, United States

**Keywords:** lentil, malnutrition, biofortification, heat, combined heat-drought, crude protein, iron and zinc, grain yield

## Abstract

Lentil (*Lens culinaris* Medikus) is a protein-rich cool-season food legume with an excellent source of protein, prebiotic carbohydrates, minerals, and vitamins. With climate change, heat, and drought stresses have become more frequent and intense in lentil growing areas with a strong influence on phenology, grain yield, and nutritional quality. This study aimed to assess the impact of heat and drought stresses on phenology, grain yield, and nutritional quality of lentil. For this purpose, 100 lentil genotypes from the global collection were evaluated under normal, heat, and combined heat-drought conditions. Analysis of variance revealed significant differences (*p* < 0.001) among lentil genotypes for phenological traits, yield components, and grain quality traits. Under no stress conditions, mineral concentrations among lentil genotypes varied from 48 to 109 mg kg^−1^ for iron (Fe) and from 31 to 65 mg kg^−1^ for zinc (Zn), while crude protein content ranged from 22.5 to 32.0%. Iron, zinc, and crude protein content were significantly reduced under stress conditions, and the effect of combined heat-drought stress was more severe than heat stress alone. A significant positive correlation was observed between iron and zinc concentrations under both no stress and stress conditions. Based on grain yield, crude protein, and iron and zinc concentrations, lentil genotypes were grouped into three clusters following the hierarchical cluster analysis. Promising lentil genotypes with high micronutrient contents, crude protein, and grain yield with the least effect of heat and drought stress were identified as the potential donors for biofortification in the lentil breeding program.

## Introduction

Micronutrient malnutrition, hidden hunger, is a serious health problem affecting more than two billion people globally (1). Among the 17 essential minerals required by the human body, iron and zinc serve a multitude of biological functions (2). An inadequate bioavailability of these two elements, in commonly eaten foods, leads to serious physiological disorders, resulting in a range of health hazards including stunting and learning disabilities in children, anemia, increased morbidity, and mortality rate (3). In Sub-Saharan Africa and South Asia regions, populations living below the poverty line are at high risk of iron (Fe) and zinc (Zn) deficiencies mainly due to the predominance in their everyday diets, of cereals-based foods which are inherently low in essential micronutrients. Iron deficiency is estimated to be prevalent among 60% of the world population (4, 5), whereas zinc deficiency is widespread among 31% population (6). There are effective interventions, including diet diversification, food fortification, and nutrients supplementation, to combat micronutrient malnutrition. However, these interventions are beyond the reach of poor households due to poverty, poor distribution network, and limited access to commercially marketed fortified foods (7, 8). As a result, sustainable food approaches are essential to mitigate micronutrient malnutrition among developing countries.

Biofortification through conventional plant breeding techniques emerges as the most promising and sustainable strategy to enhance both the level of micronutrient and their bioavailability in the edible part of food crops (9). Therefore, nutritionally improved varieties hold a great promise for meeting the challenge of addressing malnutrition and attaining nutritional security worldwide. However, achieving food security will pose a serious challenge due to the rising global population, from 7.7 billion currently to 9.7 billion in 2050 (10). In addition, climate change and variability have started showing its consequences on agricultural productivity and the nutritional quantity of foods, especially in rainfed dry regions.

Lentil (*Lens culinaris* Medikus) is one of the most important cool season food legume crops in the world with its cultivation in as many as 52 countries. Globally, it is grown on 6.1 million hectares, with an annual production of 6.3 million tons and productivity of 1,038 kg ha^−1^. The major lentil producing countries are Canada, India, USA, Turkey, Australia, Kazakhstan, Nepal, Russian Federation, Bangladesh, China, and Ethiopia, contributing more than 93% to the global output (11). Lentil is known for its high nutritive value. It contains a relatively high amount of protein (20–36%) and provides an affordable source of micronutrients in highly bioavailable forms. It also provides a range of important dietary compounds like carbohydrates, prebiotics, fiber, vitamins, amino acids, and antioxidants (12). Due to its high nutrient density, lentil has emerged as an excellent candidate for micronutrient biofortification (13). Several studies have shown significant genetic variation for nutritional parameters including iron, zinc, and protein content in lentil genotypes, including cultivated and wild species (14–16). Under the HarvestPlus program, ICARDA, in collaboration with NARS partners, has developed and released many biofortified lentil varieties for cultivation in South Asia, Sub-Saharan Africa, West Asia, and North Africa (https://www.icarda.org/media/drywire/lentil-biofortification-research-fight-hidden-hunger). With climate change and variability, lentil crop encounters water and heat stresses at different growth stages, causing severe economic damage (17). The severity of damage caused by these stresses depends on timing and the intensity of the stress (18). Lentil is extremely sensitive to heat stress, especially during the flowering and seed filling stages. Temperature exceeding 32°C can restrict photosynthesis, metabolic pathways, electron flow, and respiration rate (18), which cause flower abortion, pollen infertility, and reduction in the number of pods in lentil (19, 20). These changes lead to significant losses in grain yield (20, 21). Water stress also affects plants at different growth stages, including vegetative (intermittent drought) and reproductive (terminal drought) stages. Terminal drought can suppress nearly all the processes of lentil growth and metabolism, causing heavy yield losses (19), as it reduces flower production, pod number, and seed number (22). However, very little information is available about the negative effect of heat and drought, during seed filling, on the nutritional quality of lentil. A recent study revealed that heat and drought stress adversely impacted the nutritional quality of lentil grains (23). Therefore, this study was undertaken to (a) investigate the effect of heat and drought stress on traits associated with phenology, grain yield, and nutritional quality under field conditions, and (b) assess the genetic variation for agro-morphological and quality traits in lentil genotypes.

## Materials and Methods

### Description of the Study Area

The present study was conducted at ICARDA's Marchouch experimental station (33.56°N, 6.63°W, 392 m altitude) in Morocco. This station represents the Mediterranean semi-arid environment with high precipitation during November and December. The mean annual precipitation is around 400 mm, and the mean annual temperature is 18°C. The soil is characterized by the dominance of Vertisols with a clayey texture. The pH of the soil is neutral and ranges from 6.3 to 7.9.

### Plant Material and Experimental Design

A set of 100 lentil genotypes comprising 93 germplasm accessions originating from 46 countries and seven elite lines imanating from ICARDA breeding program were evaluated in an alpha lattice design with two replications during 2016–17 cropping season. Each genotype was grown in a 2-row plot of 1 m length, with a spacing of 30 cm between rows. In each row, seeds were sown by hand at a 2-cm depth maintaining 10-cm space between plants. In total, three experiments involving the same set of genotypes were conducted side by side by manipulating the planting date and water supply, in order to impose heat and water stress at the reproductive phase. These three experiments were considered to represent three treatments, namely the normal date of planting (treatment A), late planting with irrigation at field capacity throughout the crop period (treatment B), and late planting without irrigation during the reproductive phase (treatment C), which were referred to as treatments. Treatment A resulted in optimal growing conditions (~150 mm well-distributed rainfall and below 27°C temperature) without any heat and water stress to the plants. Treatment B (planted 65 days after normal planting date with irrigation at field capacity throughout the crop duration) imposed heat stress, as the plants were frequently exposed under field conditions to a temperature above 35°C during the reproductive phase.

In contrast, regular irrigation at field capacity avoided any water stress to the plants. Treatment C (planted 65 days after normal planting date without irrigation during the reproductive phase) imposed a combined heat and water stress. Treatment A was planted on 27 December 2016, and no irrigation was applied during the crop period as the crop received well-distributed enough rainfall at Marchouch (149.8 mm). Treatments B and C were planted on 1 March 2017. Irrigation was applied to maintain water supply at field capacity using sprinkler system throughout the crop duration in treatment B, whereas irrigation was stopped from the flowering initiation stage onward in treatment C, to impose water stress (<5 mm rainfall during the reproductive stage) in addition to the heat stress. All recommended agronomic packages of practices were followed to raise a successful crop, including nutrient and weed management throughout the growing season.

### Weather Data

**Figure 1:**
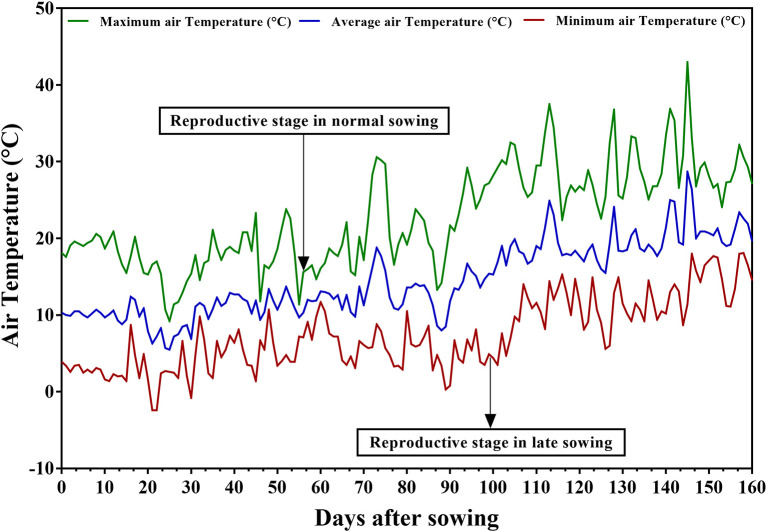
Daily maximum, minimum, and average temperatures prevalent at the experimental site at Marchouch station during the crop season of 2016–2017.

Daily minimum and maximum temperatures (°C), precipitation (mm), and relative humidity (%) were recorded throughout the crop season at the experimental site. Treatment-A experienced 9.2–23.3°C maximum and −2.4–10.7°C minimum temperatures during the vegetative stage and 11.4–30.6°C and 0.3–11.7°C during the reproductive stage (Figure 1). For Treatments B and C, maximum temperatures ranged from 13.3 to 30.6°C and minimum temperatures from 0.3 to 10.5°C during the vegetative stage and from 22.4 to 43°C and 3.5 to 18.1°C during the reproductive stage. The maximum temperatures during the reproductive stage reached the threshold level of 35°C. Therefore, late planting with irrigation at field capacity was successful in imposing heat stress during the reproductive stage of the test genotypes in treatment B, and late planting without irrigation in imposing combined stress of drought and heat in treatment C.

### Observations on Phenological and Yield Traits

Phenological observations were recorded on days to 50% flowering, days to the first pod, and days to physiological maturity on whole plot basis. At the time of harvesting, five plants were randomly selected from each plot for recording observations on yield traits namely, plant height (cm), numbers of primary, secondary and tertiary branches, numbers of total pods, filled pods, and unfilled pods, 100-seed weight (g), biological yield (g) and grain yield (g). The mean value of five plants for each trait was used for statistical analysis.

### Determination of Grain Iron and Zinc Concentrations

Seeds of each sample were ground by using a Cyclone mill (Twister, 10 mm−250 μm, Retsch). Mineral (Fe and Zn) concentrations were determined following a modified diacid protocol (24, 25). In the digestion block (QBlock series, Horiba), 0.5 g of each ground sample was placed in individual tubes and digested with 6 mL of HNO_3_ at 90°C for 60 min, followed by adding 3 mL of 30% hydrogen peroxide (H_2_O_2_) to each tube with another period of digestion of 15 min at 90°C. Then, 3 mL of 6 M hydrochloric acid (HCl) was added to each digestion tube. Finally, after the sample solutions were cooled down, the volume was adjusted to 10 mL, and then filtered. The Fe and Zn concentrations were estimated using inductively coupled plasma-optical emission spectroscopy (ICP-OES); (iCAP-7000 Duo, Thermo Fisher Scientific) at the Cereal and Legume Quality Laboratory, ICARDA, Morocco. Calibration curves for Fe and Zn were made using serial dilution from 0.1 to 10 mg L^−1^.

### Crude Protein Analysis

The nitrogen content of the samples was determined using the Kjeldahl procedure (26). In this method, the weighted seed samples of each genotype were digested with sulfuric acid followed by alkalizing the solution by adding of sodium hydroxide, and then determining the resulting ammonia by distillation into a measured volume of standard acid, the excess of which is determined by titration. Seed protein contents then were calculated using nitrogen values multiply by 6.25. For this, triplicate analyses were carried out on each sample.

### Statistical Analysis

Analysis of variance was performed using the General Linear Model (GLM). Tukey's *post-hoc* test was applied to compare the differences between the mean values. Associations among traits were assessed using the Pearson correlation coefficient (*r*). The principal component analysis was conducted to determine which traits explain most of the variation under normal, water stress and heat stress treatments and also to determine different groups of genotypes associated with major traits. Highly correlated traits with grain yield were discarded from the analysis to avoid redundant results.

Hierarchical Cluster Analysis (HCA) using Ward's method based on Euclidian distance was performed to group lentil genotypes based on mineral composition, crude protein, and grain yield. Euclidean distances were calculated after the standardization of the data. The result of the hierarchical clustering procedure was displayed graphically using a tree diagram (Dendrogram). All statistical analysis were conducted using IBM SPSS statistics version 23.

## Results

The analysis of variance revealed significant (*p* < 0.001) variation among lentil genotypes for all the traits under stressed and non-stressed conditions (Table 1). The treatment effect was also significant for the traits under investigation. The highly significant interaction effect between genotypes and treatments was detected for day to flowering, podding and maturity, grain yield, hundred-seed weight, and a number of filled and unfilled pods per plant. As for seed quality traits, the genotype × treatment interaction effect was highly significant for crude protein, iron, and zinc concentrations.

**Table 1:** Combined analysis of variance for different traits among 100 lentil genotypes at Marchouch during 2016–2017.

**Source**	**DF**	**DP**	**DM**	**PH**	**PBP**	**SBP**	**TBP**	**FPP**	**UPP**	**TPP**	**BY**	**GY**	**HI**	**HSW**	**CP**	**Zn**	**Fe**
Genotype	**	**	**	**	**	**	**	**	**	**	**	**	**	**	**	**	**
Treatment	**	**	**	**	**	**	**	**	**	**	**	**	**	**	**	**	**
Interaction	**	**	**	NS	NS	NS	NS	**	**	**	**	**	NS	*	**	**	**
Error	10.4	10.3	9.1	9.9	0.1	5.8	3.7	289.2	45.1	264.4	5.7	0.4	63.3	0.3	1.7	19.1	40.8
*R*^2^	0.98**	0.98**	0.98**	0.86**	0.68**	0.88**	0.84**	0.89**	0.80**	0.88**	0.86**	0.91**	0.80**	0.81**	0.99**	0.91**	0.92**

*, **, and SN indicate significance at 0.05 and 0.001 probability levels, and non-significant, respectively. DF, days to flowering; DP, days to first pod; DM, days to maturity; PBP, primary branches per plant; SBP, secondary branches per plant; TBP, tertiary branches per plant; PH, plant height; FPP, filled pods per plant; UPP, unfilled pods per plant; TPP, total pods per plant; BY, biological yield; GY, grain yield; HSW, hundred-seed weight; CP, crude protein content; Zn, zinc content; Fe, iron content.

### Effect Heat and Drought on Phenology and Morphological Traits

Based on each agro-morphological trait, the response of genotypes at each condition differed. The mean value of time to flower was 69 days in normal sowing while in late conditions, time to flowering was shorter, with a mean of 43 days under heat stress and 44 days under combined heat and drought stress treatments (Table 2). Similarly, days to the first pod declined by 33.4% in heat stress and 33.7% in combined heat and drought stresses. Day to maturity ranged from 100 to 120 days in normal planting, which was declined significantly in late planting with more reduction in combined heat and drought stress (23.1%) compared with heat stress (22.9%).

**Table 2:** Range and mean ± SD of phenological and morphological traits of 100 lentil genotypes under normal, heat, and heat-drought conditions.

**Trait**	**Treatment A** ** (No stress)**			**Treatment B** ** (Heat stress)**			**Treatment C** ** (Heat-drought stress)**		
	**Minimum**	**Maximum**	**Mean ± SD**	**Minimum**	**Maximum**	**Mean ± SD**	**Minimum**	**Maximum**	**Mean ± SD**
DF	53	82	68.7 ± 6.7	36	58	44.3 ± 6.6	31	55	44.2 ± 6.8
DP	62	89	75.9 ± 6.6	42	65	50.5 ± 7.4	37	62	50.3 ± 6.8
DM	100	120	110.6 ± 5.6	77	100	85.2 ± 5.5	70	96	85.0 ± 6.2
PH	21.6	38.3	31.0 ± 2.9	16.2	31.9	24.9 ± 3.6	13.4	29.4	22.1 ± 3.8
PBP	1.8	3.3	2.3 ± 0.3	2	2.7	2.4 ± 0.2	1.5	2.5	2.1 ± 0.2
SBP	9.1	28.5	16.4 ± 3.1	3.6	16.2	10.1 ± 3.0	4.7	14.7	9.6 ± 2.3
TBP	3.9	13.3	8.6 ± 2.2	1.6	7.4	4.0 ± 1.4	1.6	7.6	3.7 ± 1.1

*DF, days to flowering; DP, days to first pod; DM, days to maturity; PBP, primary branches per plant; SBP, secondary branches per plant; TBP, tertiary branches per plant; PH, plant height*.

The mean plant height was 31 cm, with a range from 21.6 to 38.3 cm in normal planting. In late sowing, plant height ranged from 16.2 to 31.2 cm in heat stress and 13.4 to 29.4 cm in heat-drought stress. The mean was 24.9 and 22.1 cm, respectively. The number of primary, secondary, and tertiary branches decreased markedly in late planting and found to decrease more in heat-drought stress. The mean of primary branches was 2.4 in normal planting. In late planting, the mean was 2.3 under heat stress condition and 2.1 under heat-drought stress condition. The secondary branches showed a marked reduction in late planting; the reduction was more significant in heat-drought stress (41.3%) than heat stress (38.1%). Similar results were found for tertiary branches, which was reduced by 53.1% in heat and by 56.8% in the combined stress.

### Effect of Heat and Drought on Grain Yield and Yield Traits

Under normal planting conditions, the mean value of biomass was 11.1 g per plant and ranged from 5.8 to 20.4 g (Table 3). On the other hand, genotypes under late planting conditions produced less biomass, which was reduced by 42.2% in heat stress and by 46.3% in combined heat-drought stress. Compared with no stress condition, the number of filled pods reduced by 55.6% under heat-drought stress and 50.7% under heat stress. Similarly, grain yield decreased by 53.5% in case of heat stress, and by 57.1% in the case of combined heat-drought stress. As for the harvest index, heat stress caused a decrease of 20.6% compared with no-stress conditions, whereas combined heat-drought stress caused a reduction of 23.2%. Hundred-seed weight ranged from 1.7 to 4.7 g, with a mean of 3.2 g in normal planting, which reduced by 26.8% under heat stress, and 34.3% under combined heat-drought stress.

**Table 3:** Range variation and mean ± SD of biomass and yield components under normal, heat, and heat-drought conditions of 100 lentil genotypes.

**Trait**	**Treatment A** ** (No stress)**			**Treatment B** ** (Heat stress)**			**Treatment C** ** (Heat-drought stress)**		
	**Minimum**	**Maximum**	**Mean ± SD**	**Minimum**	**Maximum**	**Mean ± SD**	**Minimum**	**Maximum**	**Mean ± SD**
FPP	27.8	168.6	72.2 ± 30.3	1.0	105.5	35.6 ± 25.0	0.7	74.8	32.0 ± 17.7
UPP	0.2	21.1	8.8 ± 4.8	1.2	35.3	12.7 ± 7.7	4.0	53.0	17.4 ± 9.7
TPP	32.6	175.9	81.0 ± 29.9	6.4	132.5	48.3 **±** 23.4	6.7	98.0	49.0 ± 19.0
BY	5.8	20.4	11.1 ± 2.8	2.4	18.4	6.4 ± 3.1	1.6	12.9	6.0 ± 2.4
GY	1.0	6.8	3.2 ± 1.2	0.2	3.3	1.5 ± 0.8	0.1	3.4	1.4 ± 0.7
HI	5.4	54.2	29.8 ± 10.2	3.0	44.4	23.7 ± 10.6	3.7	41.0	22.9 ± 8.9
HSW	1.7	4.8	3.2 ± 0.7	0.3	3.6	2.3 ± 0.6	0.8	2.9	2.1 ± 0.5

*FPP, filled pods per plant; UPP, unfilled pods per plant; TPP, total pods per plant; BY, biological yield; GY, grain yield; HI, harvest index; HSW, hundred-seed weight*.

### Effect of Heat Stress and Drought Stress on Grain Quality Traits

**Figure 2:**
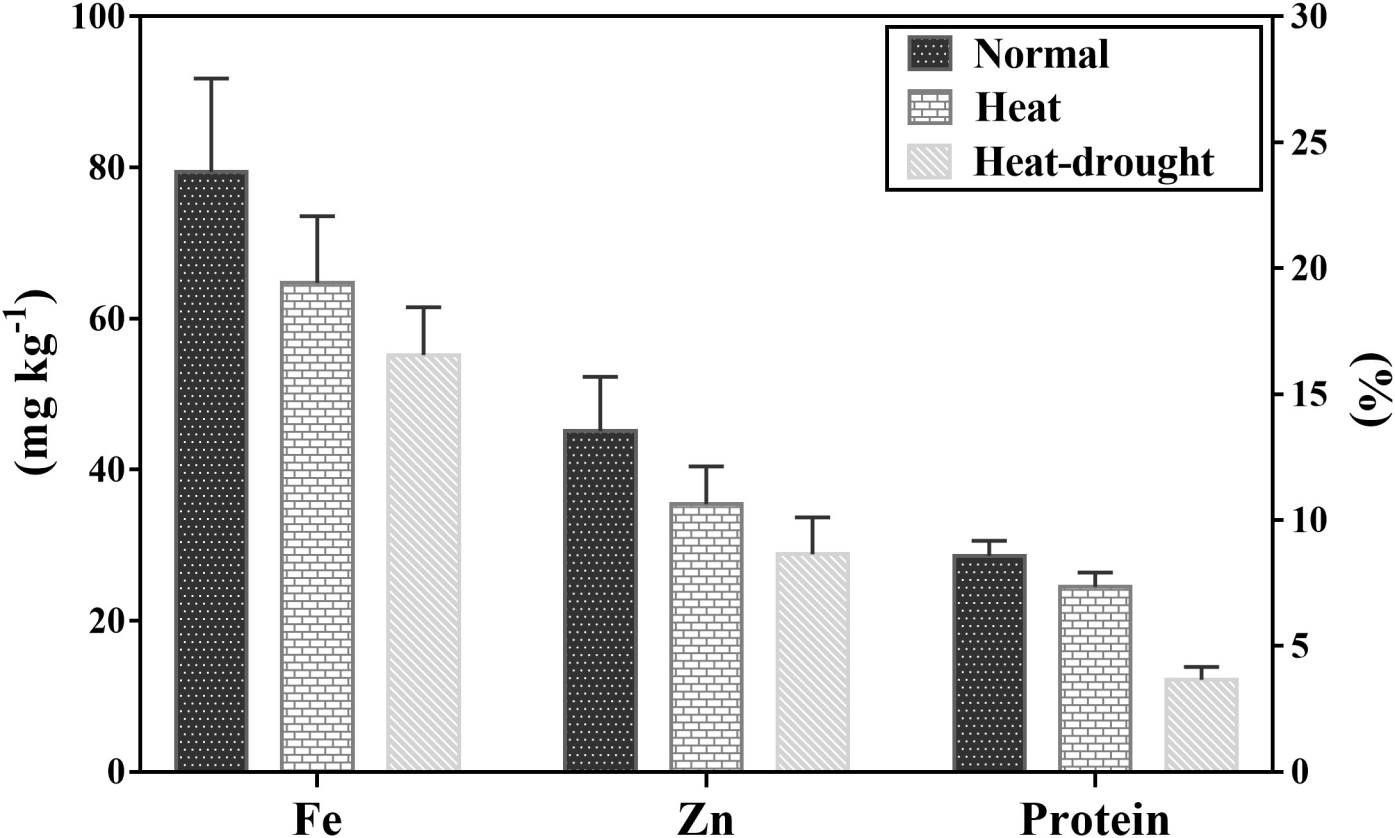
Crude protein, Fe, and Zn concentration among 100 lentil genotypes under normal, heat stress, and heat-drought stress. The value represents mean ± SD.

The mean Fe concentration was 79 mg kg^−1^ and varied from 48 to 109 mg kg^−1^ under no stress conditions (Table 4). Combined heat-drought stress significantly reduced the Fe amount by 30.4%, whereas heat stress decreased Fe concentration by 18.4%. The mean Zn concentration was 45 mg kg^−1^, and the range was 28 to 65 mg kg^−1^ under no stress conditions (Figure 2). Heat stress had a profound effect on Zn concentration with a mean 35 mg kg^−1^ and a range of 25 to 52 mg kg^−1^, which was further reduced to 29 mg kg^−1^ with a range of 18–46 mg kg^−1^ under the combined stress of heat and drought. Crude protein ranged from 22.5 to 32.0%, with a mean of 28.6% under no-stress condition. Protein content was severely affected by both stresses; combined heat-drought stress reduced crude protein by 57.2% and heat stress alone by 14.3%.

**Table 4:** Range and mean ± SD of crude protein, iron, and zinc concentrations in lentil under no stress, heat stress and combined heat-drought stress conditions.

**Trait**	**Treatment A** ** (No stress)**			**Treatment B** ** (Heat stress)**			**Treatment C** ** (Heat-drought stress)**		
	**Minimum**	**Maximum**	**Mean ± SD**	**Minimum**	**Maximum**	**Mean ± SD**	**Minimum**	**Maximum**	**Mean ± SD**
CP	22.5	32.0	28.6 ± 2.0	19.7	28.3	24.5 ± 1.9	8.5	19.4	12.2 ± 1.7
Zn	31	65	45 ± 7.2	25	52	35 ± 5.0	18	46	29 ± 4.9
Fe	48	109	79 ± 12.4	46	92	65 ± 8.8	37	76	55 ± 6.3

*CP, crude protein content; Zn, zinc content; Fe, iron content*.

### Association Between Seed Yield and Other Agro-Morphological Traits

The coefficients of correlation between seed yield and other studied traits under normal and late conditions are shown in Tables 5, 6. Seed yield showed significant positive correlations with biological yield (*r* = 0.37; *p* < 0.01), harvest index (*r* = 0.73, *p* < 0.01), number of filled pods per plant (*r* = 0.70; *p* < 0.01), number of total pods per plant (*r* = 0.71; *p* < 0.01) and 100-seed weight (*r* = 0.76; *p* < 0.01) under no stress conditions. No correlation of seed yield existed with nutritional quality traits, including protein, Fe and Zn concentrations. There existed a significant positive correlation between iron and zinc concentrations (*r* = 0.28; *p* < 0.01).

**Table 5:** Correlation coefficients between different trait combinations based on 100 lentil genotypes under no stress conditions.

	**PBP**	**SBP**	**TBP**	**FPP**	**UPP**	**TPP**	**BY**	**GY**	**HI**	**HSW**	**CP**	**Zn**	**Fe**
PH	0.02	0.02	0.09	0.11	0.13	0.13	0.45^**^	0.17	−0.12	0.35^**^	−0.06	0.20^*^	−0.03
PBP		0.40^**^	0.26^**^	−0.05	0.17	−0.02	0.27^**^	0.06	−0.11	0.01	−0.17	−0.08	−0.06
SBP			0.76^**^	0.24^*^	−0.12	0.23^*^	0.50^**^	0.17	−0.17	0.03	−0.03	0.12	−0.09
TBP				0.21^*^	−0.10	0.20^*^	0.44^**^	0.14	−0.18	0.05	−0.00	0.10	−0.08
FPP					−0.16	0.99^**^	0.40^**^	0.70^**^	0.40^**^	0.52^**^	0.02	−0.02	−0.01
UPP						−0.01	0.06	0.01	0.02	−0.07	−0.04	0.10	0.14
TPP							0.42^**^	0.71^**^	0.40^**^	0.51^**^	0.02	−0.00	0.01
BY								0.37^**^	−0.30^**^	0.33^**^	0.01	0.05	−0.20^*^
GY									0.73^**^	0.76^**^	0.12	0.02	0.09
HI										0.52^**^	0.10	0.00	0.23^*^
HSW											0.15	0.08	0.11
CP												−0.26^*^	0.03
Zn													0.28^**^

*PBP, primary branches per plant; SBP, secondary branches per plant; TBP, tertiary branches per plant; PH, plant height; FPP, filled pods per plant; UPP, unfilled pods per plant; TPP, total pods per plant; BY, biological yield; GY, grain yield; HI, harvest index; HSW, hundred-seed weight; CP, Crude protein content; Zn, zinc content; Fe, iron content; HI, harvest index*.

* *Correlation is significant at the 0.05 level*.

** *Correlation is significant at the 0.01 level*.

**Table 6:** Correlation coefficients between different trait combinations based on 100 lentil genotypes grown under heat (above diameter) and combined heat-drought stress (below diameter) conditions.

	**PBP**	**SBP**	**TBP**	**FPP**	**UPP**	**TPP**	**BY**	**GY**	**HI**	**HSW**	**CP**	**Zn**	**Fe**
PH	0.22^*^	0.45^**^	0.50^**^	0.40^**^	−0.04	0.44^**^	0.59^**^	0.39^**^	−0.03	0.24^*^	0.01	0.20	0.14
PBP	1	0.51^**^	0.45^**^	0.22^*^	0.11	0.26^**^	0.34^**^	0.12	−0.20^*^	0.06	−0.04	0.20	0.06
SBP	0.32^**^	1	0.86^**^	0.48^**^	−0.05	0.52^**^	0.71^**^	0.37^**^	−0.25^*^	0.10	0.07	0.24^*^	0.07
TBP	0.24^*^	0.81^**^	1	0.43^**^	−0.08	0.44^**^	0.71^**^	0.35^**^	−0.31^**^	0.12	0.06	0.13	0.05
FPP	0.14	0.42^**^	0.41^**^	1	−0.35^**^	0.91^**^	0.75^**^	0.87^**^	0.41^**^	0.52^**^	0.10	0.18	0.22^*^
UPP	0.09	0.20^*^	0.13	−0.11	1	−0.03	−0.21^*^	−0.43^**^	−0.36^**^	−0.26^*^	−0.07	−0.09	0.00
TPP	0.18	0.50^**^	0.45^**^	0.87^**^	0.40^**^	1	0.73^**^	0.76^**^	0.30^**^	0.45^**^	0.05	0.16	0.20^*^
BY	0.38^**^	0.74^**^	0.73^**^	0.53^**^	0.07	0.53^**^	1	0.64^**^	−0.12	0.28^**^	0.06	0.16	0.23^*^
GY	0.06	0.32^**^	0.32^**^	0.82^**^	−0.25^*^	0.64^**^	0.57^**^	1	0.61^**^	0.65^**^	0.20	0.09	0.18
HI	−0.28^**^	−0.26^**^	−0.23^*^	0.46^**^	−0.31^**^	0.27^**^	−0.20^*^	0.59^**^	1	0.52^**^	0.17	−0.01	0.02
HSW	0.12	0.20	0.16	0.44^**^	−0.19	0.31^**^	0.30^**^	0.49^**^	0.24^*^	1	−0.02	0.01	0.16
CP	0.06	−0.01	−0.07	−0.03	−0.11	−0.09	0.02	−0.07	−0.05	0.14	1	−0.03	−0.12
Zn	0.01	−0.07	−0.01	−0.23^*^	0.13	−0.15	−0.19	−0.17	−0.12	−0.01	0.01	1	0.26^*^
Fe	−0.14	−0.07	−0.05	−0.07	0.13	0.00	−0.10	0.05	0.14	0.00	0.11	0.36^**^	1

PBP, primary branches per plant; SBP, secondary branches per plant; TBP, tertiary branches per plant; PH, plant height; FPP, filled pods per plant; UPP, unfilled pods per plant; TPP, total pods per plant; BY, biological yield; GY, grain yield; HI, harvest index; HSW, hundred-seed weight; CP, Crude protein content; Zn, zinc content; Fe, iron content; HI, harvest index.

* *Correlation is significant at the 0.05 level*.

** *Correlation is significant at the 0.01 level*.

Under stress conditions, grain yield showed a significant positive correlation with plant height, biomass, and harvest index in both heat and combined heat-drought stresses (Table 6). Grain yield was positively correlated with secondary and tertiary branches in both treatments (*p* < 0.01). A significant positive correlation was obtained between grain yield and the number of filled pods per plant in both heat and combined heat-drought stresses; the coefficient of correlation was *r* = 0.87 and 0.82, respectively. Similar to the normal planting, the correlation coefficient of grain yield and 100-seed yield was highly significant and positive under both heat and heat-drought treatment (*r* = 0.65 and 0.49; *p* < 0.01).

**Figure 3:**
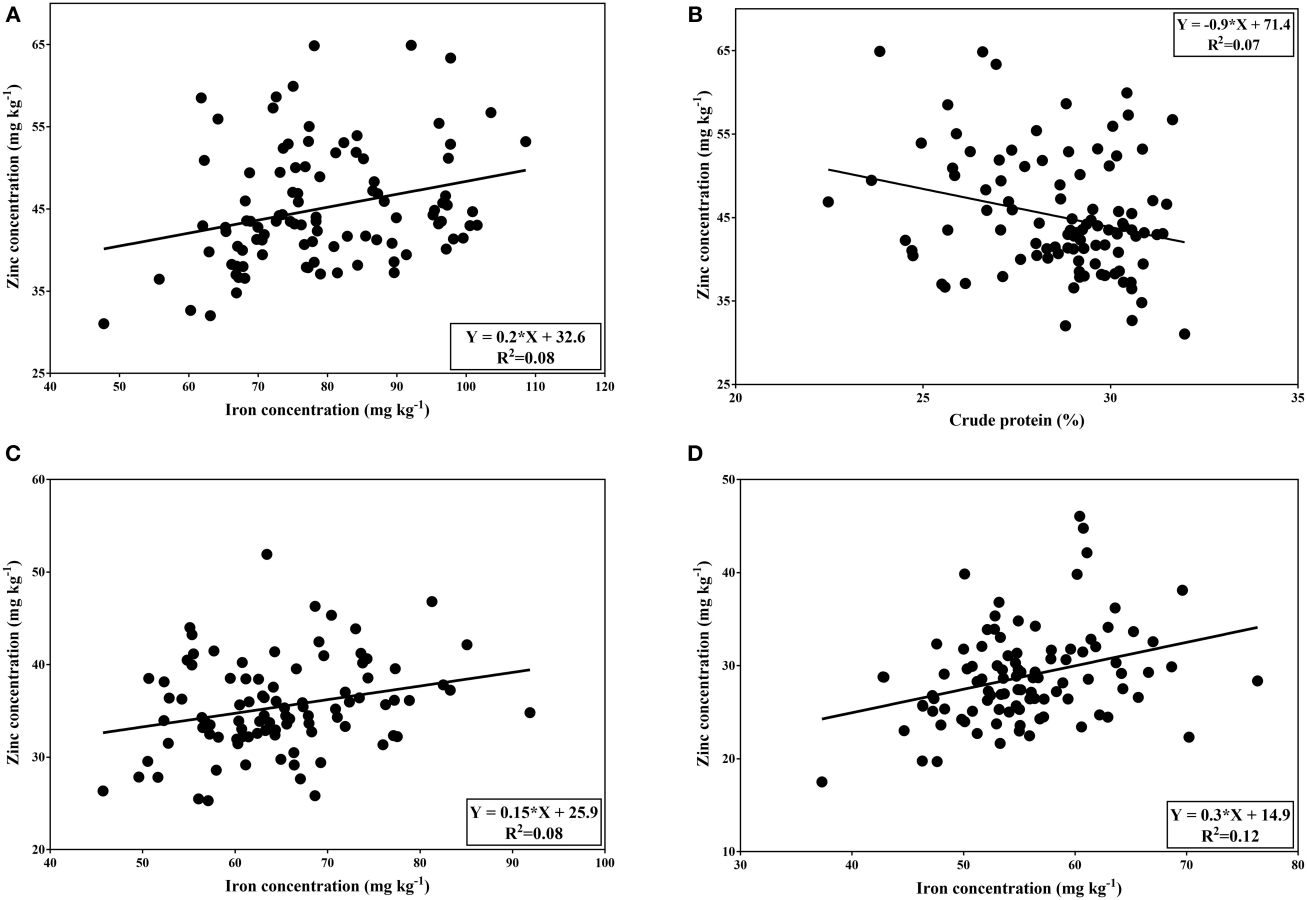
Correlation between Fe and Zn content **(A)** and between Zn content and crude protein **(B)** of tested genotypes under normal condition. Correlation between Fe and Zn concentrations under heat stress **(C)** and combined heat-drought stress **(D)**.

In both normal and late planting conditions, seed yield showed no significant correlation between seed mineral contents and crude protein contents. A significant positive correlation was demonstrated between iron and zinc concentrations in normal planting. The correlation was relatively low (*r* = 0.28; *p* < 0.01). Similarly, a significant positive correlation was found between iron and zinc concentrations under both heat and combined heat-drought treatment (*r* = 0.26; *p* < 0.05 and *r* = 0.36; *p* < 0.01, respectively). On the other hand, the correlation coefficient between zinc and crude protein was significant and negative (*r* = −0.26; *p* < 0.05) in normal condition (Figure 3).

### Principal Component Analysis

**Figure 4:**
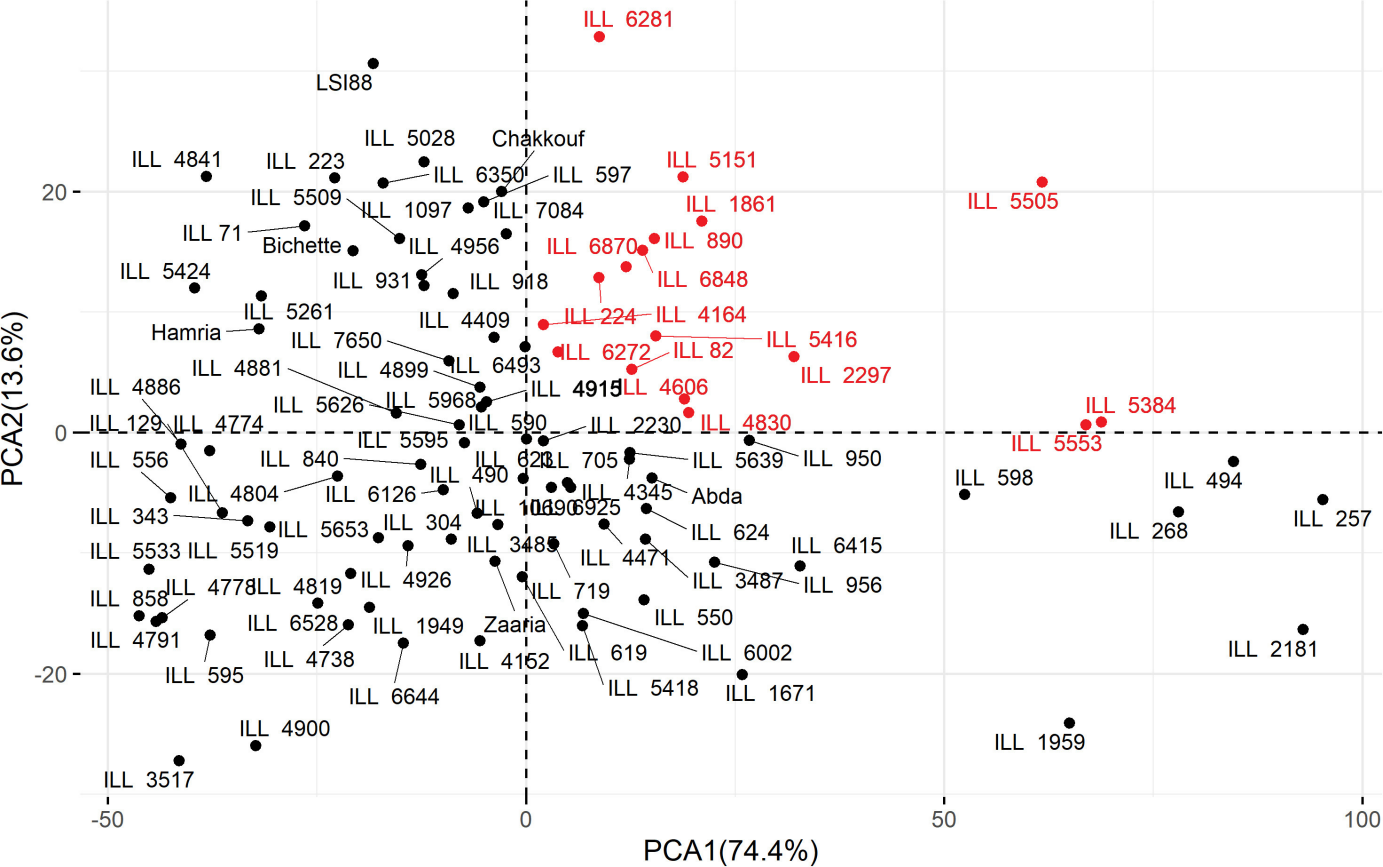
Biplot for Principal Component Analysis of 100 lentil genotypes evaluated under normal planting with no stress during 2016/2017. In Red are the lines with high Fe and high filled pods.

Principal component analysis (PCA) was used to determine which traits would differentiate the genotypes under three different treatments. Eigenvalues of the studied traits and cumulative variance are reported for each treatment in Table 7. Under stress conditions, the first two principal components explained 88% of the total variability. PCA1 and PCA2 accounted for 74.4 and 13.6% of the total variation. The PCA1 showed high association with filled pods per plant and low association with harvest index, while PCA2 was highly associated with iron content. Under heat stress treatment, the first two axes of the PCA explained 79.8% of the total variation. PCA1 accounted for 62.2% of the total variation, and PCA2 explained 17.6% of the total variation. The PCA1 was mainly associated with filled pods per plant, while PCA2 was negatively associated with unfilled pods per plant and positively with HI. Under the combined heat-drought conditions, the first two axes of the PCA explained 84.3% of the total variation with PCA1 and PCA 2, explaining 73% and 11.3% of the total variation. The PCA1 was mainly associated with filled pods per plant, while PCA2 was negatively associated with unfilled pods (−0.31) and iron content (−0.33) and positively associated with harvest index (0.88). These results revealed that the iron content contributed significantly to the variation among genotypes under normal conditions while its contribution was limited under heat and combined drought-heat stress. Biplot of PCA (Figure 4) showed that the evaluated genotypes were clustered according to iron content and number of filled pods. Under no stress conditions, the PCA1 showed clear differences among genotypes based on the number of filled pods. The right hand of this axis was characterized by genotypes with a high number of filled pods, which was highly correlated with grain yield. The PCA2 showed clear differences among genotypes based on iron content. In the upper part of the graph, all lines had higher iron concentration than the average. The upper and right part of the biplot, 17 genotypes, namely ILL 82, ILL 890, ILL 6870, ILL 6848, ILL 6281 ILL 6272, ILL 5553, ILL 5505, ILL 5416, ILL 5384, ILL 5151, ILL 4830, ILL 4606, ILL 4164, ILL 2297, ILL 1861, and ILL 224 contained higher iron and showed a higher number of filled pods compared to tested genotypes. Given the fact that iron content was highly correlated with zinc and number of filled pods with yield, the genotypes mentioned above should also be high yielding and high zinc concentration.

**Table 7:** Eigenvalues for different traits and the two major principal components with percentage variation under the different treatments.

	**No stress** ** (A)**		**Heat stress** ** (B)**		**Heat-drought stress** ** (C)**	
	**PCA1**	**PCA2**	**PCA1**	**PCA2**	**PCA1**	**PCA2**
Grain yield	0.03	0.02	0.03	0.01	0.03	0.02
Harvest index	0.15	**0.32**	**0.31**	**0.31**	0.19	**0.87**
Hundred seed weight	0.01	0.01	0.01	0.01	0.02	0.02
Filled pods per plant	**0.98**	−0.05	**0.93**	−0.25	**0.97**	−0.18
Unfilled pods per plants	−0.03	0.07	−0.15	–**0.92**	−0.12	–**0.31**
Zinc content	−0.01	**0.20**	−0.07	−0.03	0.04	−0.09
Crude protein	0.01	0.01	−0.01	0.02	0.01	0.03
Iron content	0.01	**0.93**	−0.02	−0.04	0.08	–**0.33**
Percentage variation (%)	**74.4**	**13.6**	**62.2**	**17.6**	**73.0**	**11.3**

*PCA1, First Principal Component Analysis; PCA2, Second Principal Component Analysis*.

### Identification of Potential Genotypes for Biofortification

**Figure 5:**
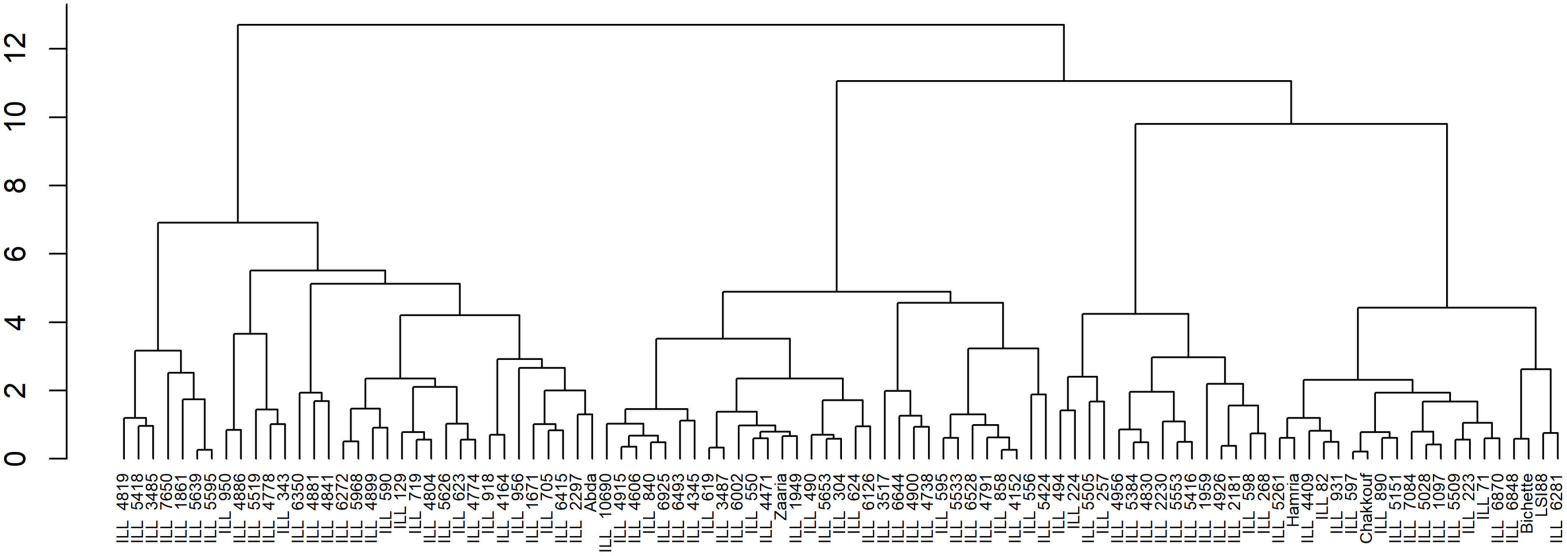
Hierarchical clustering of 100 lentil genotypes based on iron and zinc contents, crude protein, and grain yield.

Given the cluster analysis of genotypes based on grain yield, crude protein, and nutrient content, three distinct clusters were generated. The mean values of each trait in each of the three clusters are presented in Table 8. Under normal conditions, the first cluster grouped 34 lentil genotypes; this group is characterized by a high value of Fe and Zn concentrations and a moderate value of crude protein and grain yield. The second cluster contained 34 genotypes, which are characterized by a high value of iron concentration, crude protein and grain yield, and a moderate level of Zn concentration. On the other hand, the third cluster had 32 lentil genotypes. Most of the genotypes remaining in this group showed a high level of protein and a moderate level of zinc, iron, and grain yield (Figure 5).

**Table 8:** Mean value with standard deviation of grain yield, nutrient contents, and crude protein in three clusters of 100 lentil genotypes under normal, heat and combined heat-drought conditions.

	**Treatment**	**Fe**	**Zn**	**CP**	**GY**
		**Mean ± SD**	**Mean ± SD**	**Mean ± SD**	**Mean ± SD**
Cluster I	Normal	80.3 ± 11.6	50.9 ± 7.3	27.0 ± 2.2	2.8 ± 0.8
	Heat	68.1 ± 10.1	39.5 ± 4.0	23.3 ± 1.5	1.4 ± 0.7
	Heat-drought	55.7 ± 4.5	27.9 ± 4.4	12.5 ± 1.9	1.8 ± 0.6
Cluster II	Normal	86.9 ± 10.7	43.8 ± 4.7	29.4 ± 1.2	4.2 ± 1.2
	Heat	60.7 ± 75	31.8 ± 3.9	25.0 ± 2.1	0.7 ± 0.4
	Heat-drought	63.4 ± 6.0	34.3 ± 5.6	11.7 ± 0.7	0.9 ± 0.4
Cluster III	Normal	70.0 ± 8.6	40.2 ± 4.6	29.5 ± 1.3	2.6 ± 0.8
	Heat	63.9 ± 6.0	33.3 ± 2.8	25.6 ± 1.4	2.3 ± 0.6
	Heat-drought	49.6 ± 4.8	28.1 ± 4.0	12.0 ± 1.7	0.7 ± 0.4

Under the late condition, cluster analysis revealed the existence of three groups for both heat and combined heat and drought stresses. For heat stress, genotypes in the first cluster had a high level of Fe, moderate level of Zn concentration and grain yield, and low crude protein level. The second cluster was characterized by a moderate level of Zn and protein content despite reduced grain yield. Whereas, genotypes belonging to the third cluster showed high grain yield and a moderate level of Fe and crude protein. Likewise, under combined stress, genotypes in the first cluster showed moderate grain yield and Fe concentration and low crude protein. While genotype existing in the second cluster showed poor yield and low crude protein but moderate Fe and Zn concentrations. Genotypes existing in the third cluster exhibited low grain yield, crude protein, Fe, and Zn concentrations.

The top 10 lentil genotypes with a combination of high yield, high crude protein, and high micronutrient contents under normal conditions are listed in Table 9. Among the 10 top genotypes, ILL 6281, ILL 223, ILL 5151, and LSI88 are characterized by the high level of iron concentration. In comparison, the highest amount of zinc concentration was recorded for LSI88, ILL 6281, and ILL 5505. The highest crude protein was obtained for LSI88, followed by ILL 7084. As for grain yield, the greatest amount was observed for ILL 5505.

**Table 9:** Selected lentil genotypes with high micronutrient contents and their crude protein content and yield.

**Genotypes**	**Iron content** ** (mg kg^**−1**^)**	**Zinc content** ** (mg kg^**−1**^)**	**Crude protein** ** (%)**	**Grain yield** ** per plant** ** (g)**
ILL 1097	96.6	45.7	30.2	3.2
ILL 223	101.6	43.0	30.2	2.6
ILL 5028	95.2	44.3	30.3	3.6
ILL 5151	100.9	44.7	29.5	3.5
ILL 5505	97.4	51.2	30.0	5.57
ILL 5509	97.3	45.5	30.6	2.36
ILL 6281	108.6	53.2	30.9	3.3
ILL 7084	97.0	46.6	31.5	3.2
ILL 890	95.4	44.9	29.0	3.9
LSI88	103.6	56.7	31.7	3.2

## Discussion

In this study, the late planting technique was used to expose flowering and pod filling period to high temperatures. However, the confounding effects of some other environmental variables, such as relative humidity, cannot be completely controlled. The fact that heat stress occurs together with drought stress due to rapid water loss from the soil and plants (27), makes it difficult to investigate their unique impact under field conditions. Thus, to maintain heat stress conditions, high-temperature treatment was accompanied by frequent irrigation.

Lentil is particularly sensitive to high temperature at the time of flowering and seed-filling period. It may also face the combined effects of heat and drought stress, which are more severe than the individual effect (28). The temperature for optimal growth and development of lentil ranges from 18 to 30°C (29), whereas in the current study, plants were exposed to a temperature of 43°C during the reproductive stage. Moreover, exposure to high daytime temperatures (30–35°C) at this stage can have a damaging effect on many processes, including photosynthesis, metabolic pathways, electron flow, and respiration rate (19).

Our results indicate that heat stress and combined heat-drought stress, during flowering and pods filling stage, had severe impacts on growth, seed yield components, and, especially, seed nutritional quality in studied lentil genotypes. Heat stress and combined heat-drought stress accelerated all phenological growth stages. Therefore, both stresses reduced the number of days to flowering, number of days to podding, and number of days to maturity, resulting in rapid transition from vegetative to reproductive stages. Compared to the normal sowing, flowering and maturity duration were drastically reduced under both stresses, which is in accordance with earlier findings in lentil (28), chickpea (30), and common bean (31). Plant height and total plant biomass were significantly reduced by heat stress and combined stress; similar reduction has been observed in chickpea (30), faba bean (32), and common bean (33). This reduction is related to the inhibited expression of growth-related metabolism (34).

A significant reduction in the number of branches per plant was noticed under both stresses. This trait was positively correlated with grain yield under all treatments, which agrees with previous observation in lentil (35–38). Similar results were found under heat and combined heat-drought stress. Moreover, it has been shown that branch production is more important in achieving higher seed yield in lentil (39), which indicates the direct effect of the number of branches on seed yield.

Heat and combined heat-drought stress considerably decreased hundred-seed weight, which was consistent with earlier findings in lentil (23) and chickpea (30). Seed weight was a positive correlation with grain yield under both normal and late conditions; a similar kind of association was observed previously in lentil (40). Harvest index was significantly reduced under both stresses, which is consistent with the result found by Bourgault et al. (41), who reported a 16% decrease in harvest index of lentil due to the heatwave. Harvest index was also found to decrease under drought (42, 43). This index was a positive correlation with grain yield under stress and no stress condition which was in accordance with previous studies on lentil (44, 45), chickpea (46, 47). Moreover, Berny Mier Y Teran et al. (48) recently reported the existing genetic relationship between harvest index and yield in common bean and confirm the role of harvest index in the selection of both additive and epistatic effects controlling drought tolerance.

A considerable reduction in the total number of filled pods in response to heat stress and combined heat-drought stress is consistent with previous studies, where a reduction in a number of filled pods of lentil was caused by increased flower and seed abortion (49). Similar results were found in chickpea, where water deficit during pod filling period increased pod abortion (50). Several reports showed a reduction in pod set under a high-temperature regime in chickpea, which was attributed to a reduction in pollen viability and germination (51, 52).

Heat stress and heat-drought stress significantly decreased seed yield and seed weight due to the limited number of filled pods and pods losses. Reductions in grain yield varied among tested genotypes, indicating their different responses to drought and heat stress conditions. Grain yield and seed weight declined more under combined heat-drought stress than heat stress only. Our findings are supported by those found earlier (28, 53), where the impact of combined heat-drought on grain yield and grain weight was attributed to physiological and metabolic impairment of the photosynthetic components and water relations. Similar results suggested that heat alone and, in combination with drought stress, severely impaired photosynthetic function in chickpea (30).

Several studies reported that seed compound concentrations in legumes varied in response to genetic and environmental factors (54). Seed nutritional quality in lentil, including protein and mineral contents, is also significantly influenced by environmental factors and might be very sensitive to extreme weather conditions. Our results showed that iron and zinc concentrations significantly decreased under heat stress conditions, this might be attributed to the reduction of root nutrient uptake, by decreasing root biomass and metabolic rate (55) or by direct damage to roots (56). Combined heat-drought stress further decreased iron and zinc level in lentil seeds, which might be explained by the fact that decreasing water availability under drought conditions results in low micronutrient uptake, including iron and zinc (57). Furthermore, a reduced transpiration rate due to water deficit may also reduce nutrient absorption and efficiency of their utilization by the plant (58). Supporting results were recently published (23), where combined stresses intensified the impact on seed nutrients, by decreasing leaf water relations to inhibit translocation into the developing seeds. On the other hand, Smith et al. (59), reported that drought stress under field conditions had no negative impact on the accumulation of Fe and Zn in common been seeds.

Heat stress-induced significant reduction in the amount of nitrogen and, consequently, in protein content of lentil seeds. Whereas, crude protein content was dramatically reduced in response to combined heat-drought stress. Several researchers reported that drought stress is associated with changes in physiological and biochemical processes, including inhibition of protein synthesis (54). Furthermore, drought stress reduces nitrogen partitioning and fixation, resulting in a reduction in the rate of protein accumulation in the seeds (60). Our findings are consistent with early findings in lentil (23), chickpea (50), and bean (60–62), where drought stress reduced seed nitrogen and consequently seed protein content. In contrast to our findings, Hummel et al. (63) revealed that the protein level increased under drought stress conditions.

The correlation coefficient between seed mineral contents under both normal and late conditions indicates that Fe is positively correlated with Zn. In the same way, many researchers reported similar observation for Fe and Zn association in lentil seeds (12, 14, 64), chickpea (65), bean (66), and faba bean (67). In contrast, Kumar et al. (15), Thavarajah et al. (68), and Darai et al. (69) revealed no correlation between Fe and Zn contents. The positive correlations among Fe and Zn contents suggest common uptake pathways or transporters of these minerals (70, 71). These results imply that high Fe concentration can be accompanied by high Zn concentration. Under normal condition, crude protein content exhibit a low but positive correlation (*r* = 0.03) with iron content and a significant negative correlation (*r* = −0.26; *p* < 0.05) with zinc content. Our results were consistent with earlier findings in lentil (72) and chickpea (73). The negative association of zinc content with crude protein content indicates that high crude protein of lentil seeds may result in decreasing zinc concentration. This can be explained by the fact that zinc plays an important role in protein synthesis in plants (74). Moreover, it has been documented that zinc is required as a cofactor for the activity of various enzymes (75). In our study, micronutrient contents and crude protein showed a non-significant correlation with yield, making it possible to develop cultivars with high micronutrient concentrations in combination with high yield. In contrast, several studies suggested positive correlation between iron and grain yield in lentil and chickpea (65, 76).

In the present study, the multivariate analysis provides a useful mechanism for pinpointing the components that determine the variation when considering several traits simultaneously. Principal Component Analysis (PCA) indicated that most of the variation among tested genotypes was attributed to the number of filled pods and nutritional value. The identified genotypes coincided with those obtained when using the hierarchical cluster analysis (HCA). These methods were efficient in classifying lentil genotypes based on similar seed nutritional traits and grain yield, resulting in 3 clusters under both normal and stress conditions. Among the three clusters, the second cluster of the normal condition conciliates all qualities, lentil genotypes in this group are characterized by high productivity, high levels of iron concentration and crude protein, and by a moderate level of zinc concentration. Even though seed yield was markedly reduced under stress conditions, the plants were able to defend themselves to some extent from heat stress. Genotypes from clusters I and II were able to perform better and showed high grain yield per plant and a moderate level of micronutrient (Fe and Zn) and crude protein contents. However, these defenses failed under the combined heat-drought stress, resulting in a very low amount of crude protein and poor yield. Regardless, cluster I genotypes showed moderate yield and micronutrient contents.

The challenge of biofortification programs is to produce grains with high yields and superior nutritional quality, including high amounts of protein and several essential mineral elements (77). A Positive association between Fe and Zn concentrations provides the opportunity to combine these two traits in the same genotype. Furthermore, the broad-sense heritability estimate was 64 and 68% for Fe and Zn concentrations, respectively, indicating a high heritability of Fe and Zn accumulation in lentil (68, 76). Our result revealed that Fe and Zn levels seem to be yield-independent, which indicates that an increase could follow an increase in the concentration of simultaneous nutrients in grain yield, or it should at least be unaffected.

Analysis of variance results showed a high genetic variation for yield, crude protein, and micronutrient concentrations. The presence of genetic variability can be due to the diverse nature of tested genotypes. Carrying out a further analysis like cluster analysis led to the distinction of three groups of genotypes, in which yield, crude protein and iron and zinc concentrations were most responsible for their discrimination. Thus, it was possible to identify 10 lentil genotypes with a combination of high grain yield and high micronutrient concentrations, and hence, have the potential for biofortification. These genotypes can be used in genotype x environment studies to investigate whether different environments influence mineral concentrations before being included in the breeding program.

## Conclusion

The present study revealed the existence of adequate genetic variation for Fe (48–109 mg kg^−1^), Zn (31–65 mg kg^−1^), and crude protein content (22.5–32.0%) in lentil genotypes. Our results also indicate that rising temperatures alone and in combination with drought stress at the time of flowering and seed filling period severely reduced grain yield and seed quality components. We report a significant reduction in Fe (30%), Zn (35%), and crude protein (57%) levels in lentil seeds under combined heat-drought stress environments compared to 18, 22, and 14% reductions in Fe, Zn, and crude protein, respectively, under heat stress alone. The impact of combined heat-drought stress was more severe on nutritional quality compared to the mild effect of heat stress alone. This difference was more pronounced for protein compared to micronutrients. This study showed a differential response of lentil genotypes to water and heat stress and identified lentil genotypes with the least effect of stress on their grain yield and nutritional quality. These genotypes require detailed study to understand the mechanism behind no impact of heat and drought on their nutritional quality. Non-significant correlations of micronutrient and crude protein with grain yield further make it possible to develop high yielding cultivars with better micronutrient concentrations and protein. With climate change and variability, the crop is expected to encounter frequent and intense water and heat stress in rainfed dry areas. Therefore, it is essential to develop high yielding nutrient-rich varieties with the least effect of heat and water stress for sustainable lentil production.

## Data Availability Statement

The raw data supporting the conclusions of this article will be made available by the authors, without undue reservation.

## Author Contributions

HC performed the research work and prepared first draft of the manuscript. HC and KH raised experiments in fields and recorded phenotyping data. HC and AE-B analysized the seed samples for grain nutrient analysis in quality lab. NE and FM performed statistical analysis of data, SK planned and supervised research activity, and AS, FM, DT, and SK contributed in the final draft of the manuscript. All authors reviewed and approved the final version of the manuscript.

## Conflict of Interest

The authors declare that the research was conducted in the absence of any commercial or financial relationships that could be construed as a potential conflict of interest.
